# Investigation on the Effect of Dose, Frequency and Duration of Allergen Exposure on Development of Staphylococcal Infections in a Chronic Model of Canine Atopic Dermatitis

**DOI:** 10.3390/vetsci9010008

**Published:** 2021-12-28

**Authors:** Rosanna Marsella

**Affiliations:** Department of Small Animal Clinical Sciences, University of Florida, Gainesville, FL 32608, USA; marsella@ufl.edu

**Keywords:** dogs, atopic dermatitis, staphylococcal infections, canine model, allergen, *Dermatophagoides farinae*

## Abstract

Canine atopic dermatitis (CAD) is chronic and frequently complicated by Staphylococcal infections. Understanding the role of allergen dose, frequency and duration of exposure in triggering infections requires a model. Most models elicit acute inflammation and do not mimic real-life disease. Here we describe the effects of allergen exposures on development of infections in a model of chronic CAD. Diagnosis of pyoderma was based on clinical signs and consistent cytology. Study 1 evaluated the role of duration of exposure keeping the daily dose constant (25 mg/day). The one-week protocol involved three exposures, 3 days in a row. The one-month protocol involved twice-weekly challenges for 4 weeks. The three-month protocol involved twice-weekly challenges for 12 weeks. Study 2 evaluated different daily doses while keeping constant the total weekly dose (25 mg) and duration (3 weeks). Low-dose used 5 mg/day for 5 days, each week. High-dose used 12.5 mg/day twice-weekly. In Study 1, the longer the exposure, the more dogs developed pyoderma (6/9 in the three-month study, 2/9 in the one-month and 0 in the one-week). In Study 2, low-dose daily exposure caused more infections (5/8) than high-dose infrequent exposure (0/8). It is concluded that low-grade, daily exposure for a long time is most relevant for development of staphylococcal infections.

## 1. Introduction

Canine atopic dermatitis (CAD) is a chronic, relapsing inflammatory pruritic disease frequently triggered by allergens and complicated by secondary staphylococcal infections. These bacterial infections can play a significant role in aggravating the severity of the disease [1,2,3]. Due to the increased prevalence of resistant staphylococcal infections in dogs [4,5,6], it is important to understand which factors are most critical in triggering a Staphylococcal infection with the goal to minimize them. Allergen exposure in atopic dogs sensitized to a certain allergen is known to trigger clinical flares, and staphylococcal infections are frequently associated with those flares. As CAD is a complex syndrome resulting from the interaction of genetic and environmental factors, it is often difficult to do studies to address the role of specific factors in a controlled fashion when using animals in a clinical setting. For this purpose, models of spontaneously occurring disease can greatly help, as confounding factors can more easily be controlled in a research facility.

A canine model of spontaneously occurring CAD has been described in a colony of atopic Beagles [7,8]. Atopic dogs of this colony can be easily sensitized by epicutaneous exposure to allergens, and flares of disease can be induced by exposure to standardized doses of an allergen of choice while controlling factors like diet and other allergen exposure. These dogs have been proven to mimic the naturally occurring disease and thus can be very useful for studies of new therapeutic options.

The canine acute models of inflammation are typically limited to 48–96 h and do not reproduce the chronicity of the disease, which is a major characteristic of AD [9,10,11]. This approach also typically relies on either injection of a pruritogen [12] or application of allergen with some form of skin damage like tape stripping [13]. The purpose of the present study is to report on a model of chronic CAD. In this model dogs can be epicutaneously challenged with allergen in a long-term fashion mimicking more a real-life situation in which dogs encounter the allergens for more extended periods of time. This model does not involve any injection of a pruritogen or application of large amounts of allergen under occlusion. Sensitization in these dogs does not involve injections or tape stripping. The availability of a chronic model of AD is important to address the question of secondary bacterial infections. In this prospective study we aimed to evaluate which aspects of allergen exposure are most relevant in the development of a bacterial infection. The factors evaluated included the following: (1) duration of allergen exposure; (2) daily dose of allergen; and (3) frequency of allergen exposure.

## 2. Materials and Methods

All the animal procedures in these studies were approved by the Institutional Animal Care and Use Committee of the University of Florida.

### 2.1. Dogs

Nine atopic dogs belonging to an atopic colony previously validated were used for Study 1, and eight dogs were available for Study 2 (one dog had been adopted out). At least one month wash out was observed between rounds of allergen challenges. Dogs that were diagnosed with pyoderma were treated topically with twice weekly benzoyl peroxide shampoo until resolution of their pyoderma before they observed the required wash out period from allergen exposure. If pyoderma was too severe for topical therapy only, systemic antibiotic therapy would be prescribed.

### 2.2. Protocols for Allergen Challenges

#### 2.2.1. Study 1: Evaluation of Duration of Allergen Exposure

**Figure 1:**
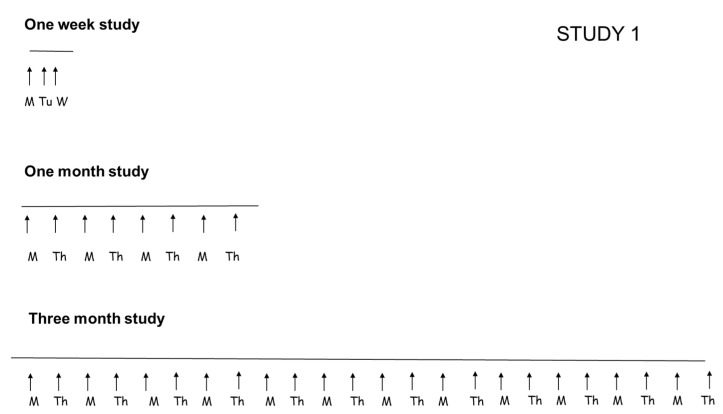
Protocol of allergen challenges in Study 1 aimed to assess the effect of duration of allergen exposure. The same allergen dose was used for each dog (25 mg of house dust mite) in the three protocols, but different duration and number of challenges was done in the three studies. In the one week study, the dogs were challenged Monday, Tuesday and Wednesday (M, Tu, W). In the one month and the three-month studies, the dogs were challenge Monday and Thursday (M, Th) of each week.

To evaluate the importance of duration of allergen exposure, three protocols of allergen challenge were compared. In all these protocols, the daily dose of allergen used was always the same (25 mg of *Dermatophagoides farinae* solution/dog/challenge) but the duration of the study and number of allergen challenges varied (Figure 1). Allergen was purchased from Stallergenes Greer, NC, USA and was applied epicutaneously on the inguinal area of the dogs, mixed with saline, without any occlusion. No prior tape stripping was done.

In the one-week study, dogs were exposed to allergen 3 days in a row (Monday, Tuesday and Wednesday). In the one-month study, dogs were exposed to allergen twice-weekly (Monday and Thursday) for a total of 4 weeks. In the three-month study, dogs were exposed to twice-weekly challenges (Monday and Thursday) for a total of 12 weeks.

#### 2.2.2. Study 2: Evaluation of the Effect of Different Daily Doses (While Keeping Constant the Total Weekly Dose of Allergen and the Overall Duration of the Study)

**Figure 2:**
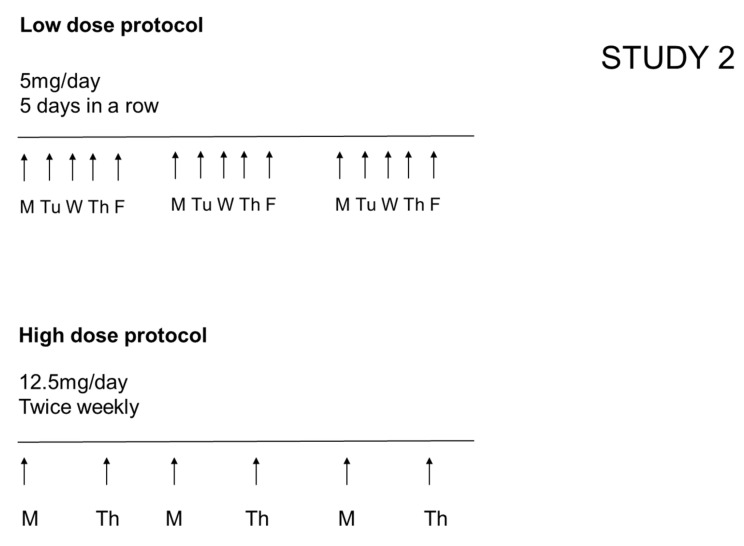
Protocol for allergen challenge in Study 2 aimed to address the effect of daily dose of allergen while keeping the weekly dose the same (25 mg/dog) and the duration the same (3 weeks). In the low dose protocol, dogs were exposed to house dust mites every day of the week, 5 days in a row, Monday, Tuesday, Wednesday, Thursday and Friday (M, Tu, W, Th, F). In the high dose protocol, dogs were challenged twice weekly with 12.5 mg of allergen, Monday and Thursday (M, Th).

In the low dose protocol, a dose of 5 mg/dog/day was used, 5 days/week (total weekly dose of 25 mg/dog) for a total duration of the study of 3 weeks. In the high dose protocol, 12.5 mg/dog/day was used twice weekly for a total of 3 weeks (Figure 2).

### 2.3. Clinical Assessment of Severity of Dermatitis and Development of Pyoderma

Dogs were clinically assessed every day of an allergen challenge, 3 h after the challenge. The same investigator made all the assessments. Severity of dermatitis was scored using a validated scale (CADESI-03 [14]). Development of pyoderma was assessed at the end of the challenges based on clinical signs such as crusted papules, pustules and collarettes. When pustules or crusted papules were present, they were used for cytology. Detection of neutrophils with intracellular cocci and/or degenerated neutrophils with extracellular cocci were considered consistent with a clinical diagnosis of secondary staphylococcal infection. 

### 2.4. Statistical Analysis

A mixed model analysis was used for the dermatitis scores to evaluate the effect of time, group and group x time interaction (JMP Pro software, Cary, NC, USA). *p* < 0.05 was considered significant.

## 3. Results

### 3.1. Study 1. Evaluation of Duration of Allergen Exposure (While Keeping the Dose of the Allergen Challenge the Same)

**Figure 3:**
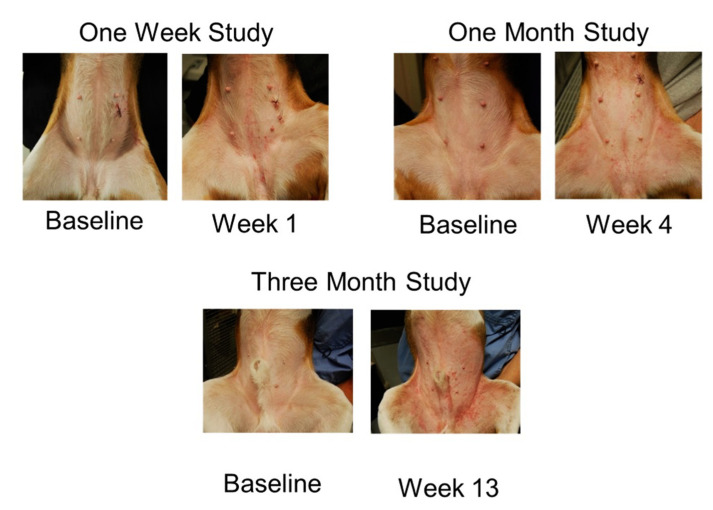
Examples of dogs’ presentations at baseline (prior to any allergen challenge) and at the end of the allergen challenges.

**Figure 4:**
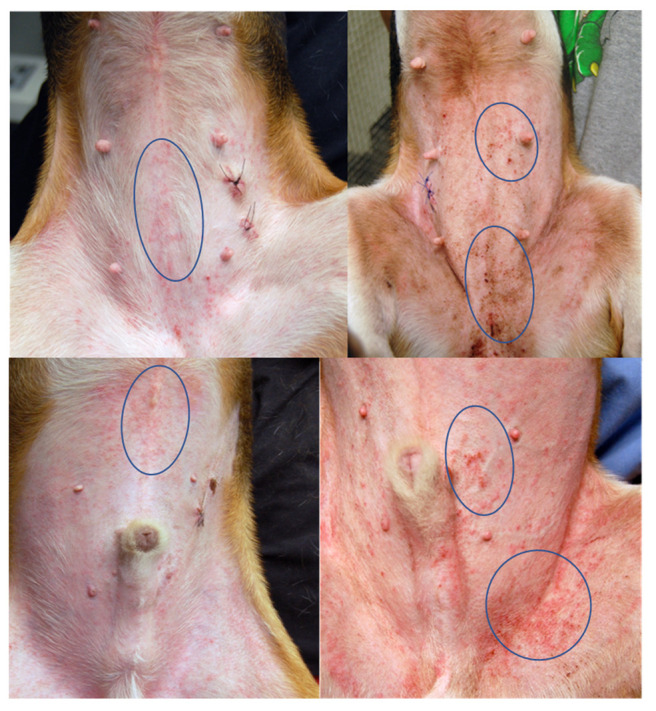
Close up of images of lesions of atopic dermatitis (**left**) and atopic dermatitis with superimposed staph infection (**right**). Clinically speaking, the papules that develop after HDM exposure in this model are small and of fairly even size. When a staphylococcal infection develops, the papular eruption becomes larger, and exudative lesions develop, which are then covered with crusts. The circles are areas of example of the differences. Biopsies were taken for reasons not related to this study.

**Figure 5:**
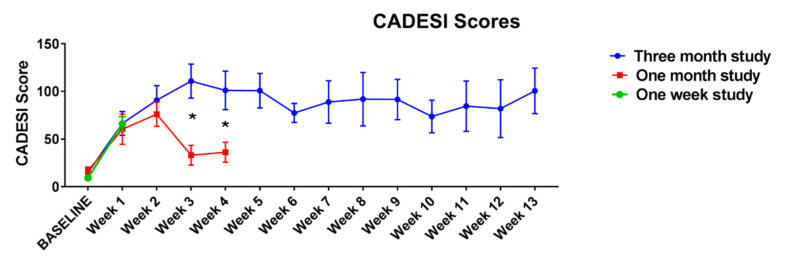
Clinical scores (Canine Atopic Dermatitis Extent and Severity Index [CADESI] means and standard deviations) of the first set of studies. When comparing the one-month study and the three-month study (week 0–4), a significant effect of the group was noted (two groups being different at weeks 3 and 4, *p* < 0.01, as indicated by the asterisk).

At the end of the challenges, 6/9 dogs (66.6%) developed pyoderma in the 3-month protocol, 2/9 (22.2%) in the one-month protocol and none in the one-week protocol. Dogs developed erythema, macules and papules (Figure 3) and excoriations as result of allergen exposure and secondary pruritus. Figure 4 shows closeup of clinical lesions to illustrate the clinical differences between house dust mite triggered atopic dermatitis lesions in this model and lesions that had developed a staphylococcal component. In dogs with a staphylococcal component, dirty brown scaling was evident, and exudative lesions and crusted lesions were present. As papules were listed in the (Canine Atopic Dermatitis Extent and Severity Index (CADESI) scores, the increase in scores was, in some dogs, at the end of the challenges driven by the over imposed infection. Clinical scores increased over time in the course of the allergen challenges, but in the one-month protocol a decrease was noticed at weeks 3 and 4, while in the three-month protocol the scores peaked at week 3 and remained at that level for the rest of the challenges (Figure 5).

### 3.2. Study 2. Evaluation of the Effect of Different Daily Doses (While Keeping Constant the Total Weekly Dose of Allergen and the Overall Duration of the Study)

**Figure 6:**
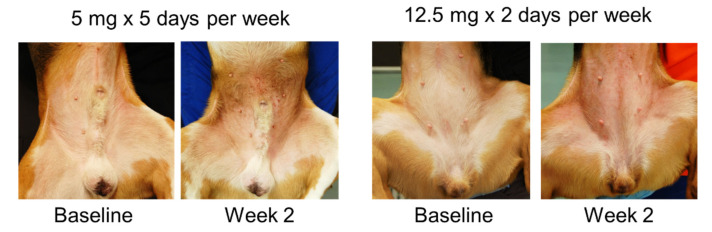
Examples of dogs’ presentations in the two groups at baseline and after two weeks of challenges.

**Figure 7:**
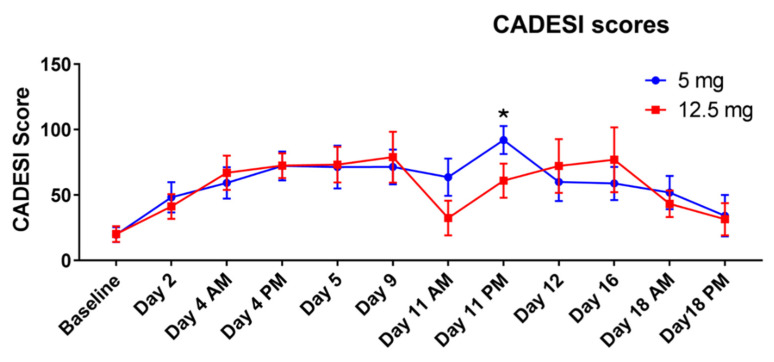
Clinical scores (means and standard deviation) of the dogs undergoing the low dose protocol (5 mg/dog/day, 5 days/week) and the high dose protocol (12.5 mg/dog twice weekly). On days of allergen challenge, dogs were evaluated in the morning (AM, prior to the allergen exposure) and in the afternoon, 6 h after exposure (PM). The asterisk indicates when the scores of the two protocols were different.

At the end of the challenges, 5/8 dogs (62.5%) developed pyoderma with the low dose protocol, while none developed pyoderma when undergoing the high dose protocol. Dogs developed erythema and papules as reactions to allergen exposure (Figure 6). The scores of the dermatitis were not statistically different between the two groups for the majority of the study. The only difference was detected on day 11 (Figure 7) when the group challenged with the lower daily amount of allergen had higher scores.

## 4. Discussion

This study focused on the duration and dose of allergen exposure to investigate whether these factors may play a role in triggering pyoderma in atopic dogs in a research setting where other factors like other allergens and diet are controlled. It was found that low-grade, daily exposure to house dust mites for long periods of time are most relevant for development of staphylococcal infections. Indeed, Study 1 showed that the longer the exposure, the more dogs developed pyoderma (6/9 in the three-month study, 2/9 in the one-month and none in the one-week), and Study 2 showed that low-dose daily exposure caused more infections (5/8) than high-dose infrequent exposure (none).

These findings are consistent with what is observed in real life in where low grade, daily exposure to house dust mites for long periods of time is what happens to pets that live indoors. House dust mite allergic indoor dogs have ample opportunity to have daily, low grade exposure for extended periods of time. Mechanisms by which low grade chronic exposure facilitates the development of a bacterial infection can range from damage in the skin barrier due to proteolytic activity of the mites [15] to promotion of low-grade inflammation which promotes dysbiosis and makes the skin more vulnerable to staphylococcus infection [16]. *Dermatophagoides farinae* has been shown to be the most common mite found in the dust collected in homes with dogs in the USA, and dog beds older than one year were the ones reported to have the highest amounts of *D. farinae* [17].

Based on the results of these protocols of allergen challenge, it could be speculated the atopic patients can cope with larger amounts of allergen if they have some time between exposures so that the skin can recover. As the allergen was not applied under occlusion in this study, the dogs were free to lick the inguinal area and remove the dust from their skin. Thus, even a larger amount of allergen less frequently was not as impactful as the daily application of dust mites when the skin did not have sufficient time to recover from daily stimulation.

The colony used for this study is different from other models of canine AD described in the literature. These models reported acute challenges involving application of allergen, sometimes under occlusion or mixed with an oil base [9,11,18,19]. For this reason, we cannot really compare our type of study with other studies in the literature.

This study has several important limitations. The number of dogs used in the study was low, and there were no control dogs (e.g., normal dogs challenged with house dust mite). The challenge of normal dogs had been done at the time of the validation of this model [8] but not in the study here described. The initial validation [8], however, did not involve weeks and weeks of challenges but focused more on the effect of a higher dose (50 mg of *D. farinae* for 3 days in a row). In the validation study, the normal dogs did not develop any reaction, while the atopic Beagles of the colony developed severe erythema, papules and pruritus and so did the other dogs with naturally occurring disease used as positive controls [8].

It is interesting to note that in Study 1, the scores of the dogs in the one month and three-month allergen study were noticeably different at week 3 and 4. These were always the same dogs, and they had observed standard wash out so that at baseline the scores were similar for each protocol. The reason for these differences is not completely understood. It is possible that, since the three-month study had occurred after the one-month study, the dogs had been primed by the previous rounds of challenges and responded more severely in the three-month study. This increased responsiveness could have also played a role in explaining their vulnerability in the development of secondary infections.

It is important to notice that the dogs developing pyoderma were not always the same dogs. In the system used to score severity of dermatitis, some increase of the scores was driven by the secondary staphylococcal component as it was not possible to separate exactly what part of the dermatitis was pure AD and what also had a secondary staphylococcal component. It is also true that staphylococcus aggravates AD even in the absence of a full-blown pyoderma; thus, it is very likely that for some dogs the scores were a combination of the two diseases. None of the dogs was considered too severe to be pulled out of the challenges, and thus all dogs completed the protocols. Severity of pyoderma also was not graded in this study, and the number of dogs was so small that no conclusions could have been made from grading pyoderma. Pyoderma always only occurred in the glabrous areas, where the dogs were exposed to the house dust mite solution and it was never generalized. It is possible that if they had been left untreated that a more generalized pyoderma could have occurred, as it is observed in many of atopic dogs in the clinics.

## 5. Conclusions

It is concluded that low-grade, daily exposure for long periods of time is most relevant for development of staphylococcal infections.

## Data Availability

Data can be available upon reasonable request.
